# Review: Mechanochemistry of the kinesin‐1 ATPase

**DOI:** 10.1002/bip.22862

**Published:** 2016-05-20

**Authors:** R. A. Cross

**Affiliations:** ^1^ Centre for Mechanochemical Cell BiologyWarwick Medical SchoolCoventryCV4 7ALUK

**Keywords:** kinesin, ATPase, mechanochemistry

## Abstract

Kinesins are P‐loop NTPases that can do mechanical work. Like small G‐proteins, to which they are related, kinesins execute a program of active site conformational changes that cleaves the terminal phosphate from an NTP substrate. But unlike small G‐proteins, kinesins can amplify and harness these conformational changes in order to exert force. In this short review I summarize current ideas about how the kinesin active site works and outline how the active site chemistry is coupled to the larger‐scale structural cycle of the kinesin motor domain. Focusing largely on kinesin‐1, the best‐studied kinesin, I discuss how the active site switch machinery of kinesin cycles between three distinct states, how docking of the neck linker stabilizes two of these states, and how tension‐sensitive and position‐sensitive neck linker docking may modulate both the hydrolysis step of ATP turnover and the trapping of product ADP in the active site. © 2016 Wiley Periodicals, Inc. Biopolymers 105: 476–482, 2016.

## INTRODUCTION

Eukaryotic cells contain networks of microtubules that serve as railways for the motor‐driven transport of cellular components. Together with the dyneins, kinesins are the molecular engines for this cellular railway. Most kinesins haul molecular cargo directionally along microtubules, but some are specialized to control the assembly dynamics of their microtubule tracks, and a few can do both. These two distinct activities, hauling cargo along microtubules and biasing subunit exchange at microtubule tips, are linked by a common thread, the generation and sensing of mechanical force in the kinesin active site. Because the active site chemistry generates force, its chemical equilibria are in turn sensitive to external mechanical force. This connectedness, termed mechanochemical coupling, works reciprocally—it allows chemical kinetic events in the kinesin active site to be harnessed to drive a larger scale conformational cycle that in turn can do substantial work, and it also allows external forces sensed by the motor to influence the chemical kinetics of ATP turnover. Recently, the force‐generating and force‐sensing mechanisms of the kinesin active site have come much more clearly into focus. Here I review how the kinesin active site processes ATP and outline how its conformational programme can both drive and be driven by the larger‐scale conformational programme of the motor as a whole. To do this I focus largely on kinesin‐1, the best studied kinesin.

## BINDING STATES

ATP turnover cycles the kinesin motor domain between strong and weak binding states. Strong states are defined by their ability to bind microtubules stably and stereospecifically, and to hold force. Weak states are defined by their tendency to detach from microtubules. Kinesin in solution is usually in a weak K.ADP state (see later). The binding of this K.ADP state to microtubules accelerates ADP release, converting the motor domain to its empty (apo) state, which is strong binding. The subsequent binding of MgATP triggers a clamshell closure of the two switch regions, which flank the active site, creating a new K.ATP conformational state. This state is also strong. Switch closure facilitates ATP hydrolysis, generating the K.ADP.Pi state, which is also strong. Phosphate release then regenerates the weak K.ADP state^1^ which shows the greatest tendency to detach from the microtubule. This mapping of nucleotide states to binding states appears to be broadly the same for all kinesins so far examined, although the rates of ATP turnover, the fraction of time spent in each state and the effects of microtubules and unpolymerized tubulin on the rate constants for transitions vary widely—for example in kinesin‐13, a depolymerase kinesin, the K.ADP state is still the detaching state, but the binding of unpolymerized tubulin seems to be required for hydrolysis.^2^, ^3^

## THE SWITCH MECHANISM

**Figure 1:**
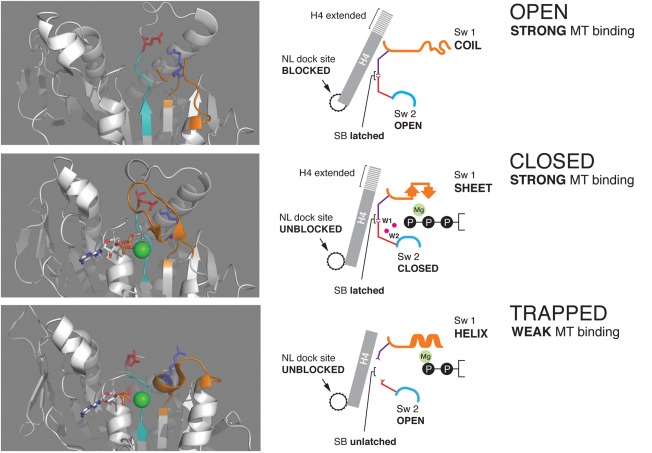
Three states of the kinesin active site switch machinery. In the OPEN state (above), the nucleotide is gone and Sw1 (orange) is unstructured. Sw2 (cyan) is retracted. In the structure shown (part of 4LNU,^8^ human apo kinesin‐tubulin complex) the critical latch bridge linking Sw1 to Sw2 (SSRSH to LAGSE) is intact. In the CLOSED state (center; 4HNA, human kinesin‐tubulin complex in ADP AlF_4_,^38^), ATP has bound and both switches have closed. The critical salt bridge linking the switches is latched and the active site is hydrolytically‐competent, with the lytic (W1) and proton‐acceptor (W2) water molecules in position for nucleophilic attack on the gamma phosphate. Tight coordination of the Mg^2+^ ion (see text) locks the nucleotide in place and displays the gamma phosphate into the catalytic center. The Sw1 is configured as two short antiparallel beta sheets. In the TRAPPED state (below; 1BG2, human kinesin‐1.ADP complex^20^), the Sw1 refolds into a helix and part of the Sw1 loop is recruited to extend H3. The critical salt bridge is broken. The Mg^2+^ ion remains in place, locking in the ADP, but its coordination changes (see text). In the OPEN and CLOSED states, the H4 helix (the Sw2 helix) is extended, and the kinesin motor domain is in a strong binding state that forms a stable complex with tubulin. In the TRAPPED state of the switches, H4 shortens, Sw1 retracts and the motor domain is in a weak binding state that detaches from tubulin. Graphics prepared using Pymol (www.schrodinger.com).

Kinesins are P‐loop NTPases and, as with other family members, the P‐loop is substantially rigid and invariant in the different structures so far visualized. When ATP docks against the P‐loop, the switch regions close on it and a salt bridge forms to connect them, linking the conserved Glu236 of switch 2 (Sw2; DLAGSE) and the conserved Arg203 of switch 1 (Sw1; SSRSH) (throughout, residue numbers refer to human kinesin‐1). When Sw1, shown in orange in Figure 1, closes, the side chain hydroxyl of Ser201 (SSRSH) interacts with the γ‐phosphate, whilst the side chain of Ser202 (SSRSH) binds directly to the active site Mg^2+^. The Mg^2+^ ion is a key organizer of the active site (see below). Sw2 (cyan in Figure 1) closes on the ATP from the opposite side to Sw1 such that its conserved Gly234 (DLAGSE) binds directly to the γ‐phosphate of the ATP. With both switches CLOSED on the ATP, two key water molecules are precisely positioned to allow a nucleophilic attack on the γ‐phosphate by one of them, leading to nucleotide hydrolysis.^4^ In this CLOSED, hydrolytically competent conformational state of the active site, Sw1 has an antiparallel beta sheet conformation (Figure 1). All other states of the switches are usually just termed OPEN. In this terminology the term OPEN is a portmanteau that subsumes states with mutually opposite biochemical properties. In an earlier review,^5^ I therefore introduced a three‐state model in which the switch machinery cycles between CLOSED, TRAPPED, and OPEN states. I use this same scheme here. Each of these three states has distinct, well‐defined structural and biochemical kinetic properties, as summarized in Figure 1. Notably, the OPEN and CLOSED states are both strong, whilst the TRAPPED state is weak (Figure 1).

## THE APO (EMPTY) STATE

Kinesins typically purify with MgADP trapped in their active sites. Treatment with EDTA sequesters the Mg^2+^ and triggers rapid ADP release, but the resulting apo state then tends to denature.^6^ In complex with microtubules, the story is very different. Strong binding to microtubules activates the release of MgADP by up to three orders of magnitude,^7^ and creates a stable apo kinesin‐microtubule complex. A closely related ground state complex with unpolymerized tubulin was recently visualized by X‐ray crystallography at 2.19A resolution (4LNU, Ref. 8). In this structure, key residue–residue interactions identified by Muto and colleagues using tubulin mutagenesis^9^, ^10^, ^11^ are present. Tubulin binding stabilizes the helix alpha 4 in an extended conformation, in which it gains 2.5 helical turns compared to its solution state. The switch 1 is melted, but the critical Sw1–Sw2 latch bridge remains connected. This shows that the formation of this critical latch bridge connection is not enough in itself to structure the Sw1, despite tubulin being bound and the helix 4 being thereby lengthened. Instead, Mg‐nucleotide binding is required. Conversely, this structure also shows that nucleotide binding is not required to latch the critical salt bridge. The 4LNU structure is related to but not identical with the apo kinesin microtubule complex, because in 4LNU tubulin is in a curved configuration that differs from its conformation when built into microtubules. It seems clear that most of the kinesin–microtubule apo state interface is present in this structure, but it remains possible that extra contacts formed by the binding of apo‐kinesin to straight (polymerized) tubulin might for example strain the motor domain sufficiently to disconnect the latch bridge and entirely decouple the Sw1 and Sw2.

## NUCLEOTIDE BINDING

MgATP and MgADP both bind to the apo state of kinesin, with affinities in the low micromolar range, so that MgADP and MgATP compete to bind into the active site. For kinesin, this means that sustained, ATP‐driven directional progress is only possible when the concentration ratio of MgATP to MgADP is high (as it usually is in cells). We have recently looked into this in my own lab and find that the velocity and the stall force of kinesin‐1 are halved if the MgADP concentration is set approximately equal to the MgATP concentration (unpublished). This situation is counterintuitive and very different for example, from that of myosin—for most myosins, the affinities for MgATP and MgADP differ by three orders of magnitude. Equally surprisingly, the kinesin active site chemistry is relatively promiscuous: the open architecture of the active site allows it to process a wide range of other nucleotides and analogues.^12^ The most informative analogue has been MantATP, whose fluorescence can monitor both nucleotide binding and a conformational change associated with hydrolysis.^13^

## HYDROLYSIS

**Figure 2:**
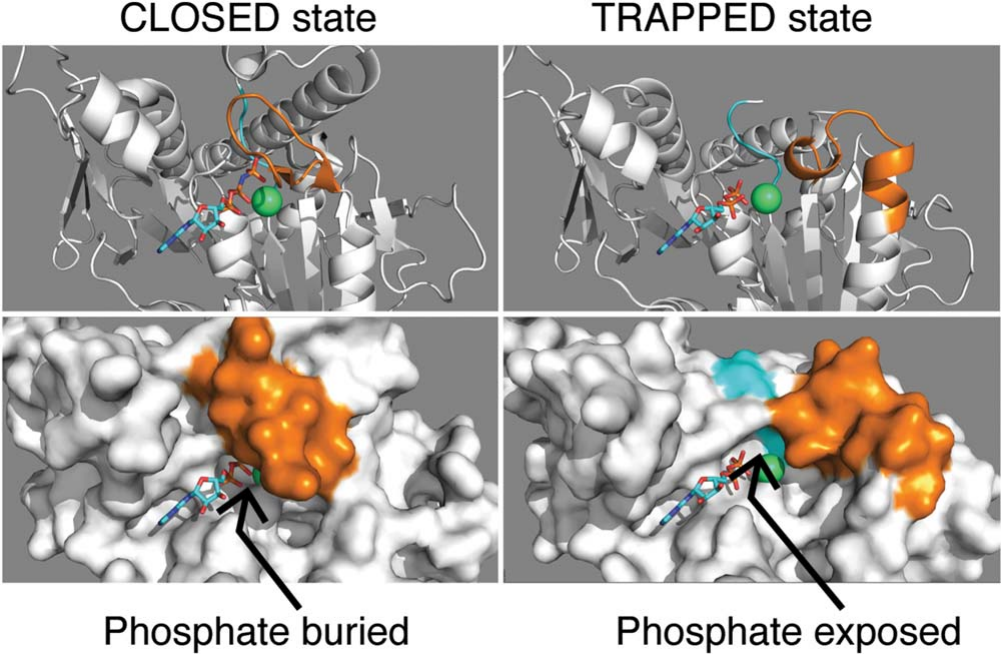
The phosphate tube. Cartoon and spacefilling views of the catalytic center, with switches colored as for Figure 1. (Left) 3HQD, kinesin‐5.AMPPNP complex^4^ (Right) 3KEN, kinesin‐5.ADP complex with STLC inhibitor.^39^ In the ATP analogue (left) the switches are in their CLOSED state and the gamma phosphate and the Mg^2+^ ion are deeply buried. In the TRAPPED state (right) the switches reconfigure themselves to open up an escape route for the phosphate.

Kinesin is a P‐loop ATPase and incoming nucleotide docks against the conserved P‐loop, which serves as a rigid template that directs the gamma phosphate of ATP into the catalytic center. The docked nucleotide is locked in place by Mg^2+^ coordination. The Mg^2+^ ion has a high charge density, allowing it to act as an electrostatic organizing center for the active site. The coordination of Mg^2+^ differs slightly between the CLOSED and TRAPPED states. In the CLOSED K.ATP state, the Sw2 and Sw1 are latched together by the critical salt bridge linking Arg203 of Sw1 (SSRSH) and Glu236 of Sw1 (DLAGSE). The gamma phosphate is engaged by the Sw2 Gly234 (DLAGSE). The Mg^2+^ is directly coordinated by Thr92 of the P‐loop (GESHGEET), by oxygens from the beta and gamma phosphates, and by Ser201 (SSRSH) of Sw1. In this state Asp231 in Sw2 (DLAGSE) makes a salt bridge to Arg190 at the top of H3.^14^ This salt bridge breaks on Pi release, as does the critical “latch” salt bridge. In the resulting TRAPPED K.ADP state, Asp231 directly coordinates the Mg^2+^ and Sw1 adopts a helical conformation (Figure 2) in which the coordination of Mg^2+^ is shared between the two conserved serines of Sw1 (SSRSH), via bridging water molecules.

Switch closure is thus driven by ATP binding and depends critically on the presence of the gamma‐phosphate. With both switches CLOSED on the ATP, two key water molecules are precisely positioned to allow a nucleophilic attack on the γ‐phosphate by one of them, leading to nucleotide hydrolysis.^4^ A two‐waters mechanism was first suggested for myosin by Onishi.^15^ In kinesins, the key water molecules are positioned specifically by the formation of the critical salt bridge that latches Sw1 to Sw2. Mutation of this salt bridge powerfully inhibits hydrolysis,^16^ as does mutation of the equivalent salt bridge in myosin.^17^

In kinesin the first (lytic) water (W1) is positioned almost exactly on‐axis with the beta‐gamma phosphate bond. It is coordinated by Sw1 Ser202 (SSRSH), by the Mg^2+^, by Sw2 Gly234 (DLAGSE) and by the second water molecule (W2). The Mg^2+^ ion thus is pivotal in hydrolysis, not simply in stabilizing and orienting the bound nucleotide, but also in organizing the hydrogen bonded network of water molecules that drives catalysis. W2 is thought to serve as the proton acceptor for the nucleophilic W1.^4^ W2 is also coordinated by Sw2 Gly234 (LAGSE), and by Sw2 Glu236 (LAGSE), which in turn makes the critical salt‐bridge with Sw1 Arg203 (SSRSH). The last major player in the hydrolysis mechanism is Sw1 Asn198, which H‐bonds to the beta‐gamma bridging oxygen, potentially increasing its electronegativity and accelerating hydrolysis.^4^

## MICROTUBULE‐ACTIVATION OF HYDROLYSIS

Microtubule binding activates the hydrolysis step of ATP turnover, by ∼ 10‐fold for kinesin‐1).^18^ Cao and colleagues^8^ argue based on their structure that neck linker docking acts as a lock on subdomain motion that stabilizes the CLOSED, hydrolytically competent state, thereby boosting ATPase activity. Importantly however, and as Cao et al. discuss, neck linker docking is not *required* for microtubule‐activation of the kinesin ATPase. Neck linker docking is initiated and to some extent driven by the annealing of a triplet of residues at the root of the neck linker (IKN) into a hydrophobic pocket formed from conserved residues in H4 and H6 and by the overlying N‐terminal cover strand.^19^

Since the first kinesin crystal structures were determined,^20^, ^21^ evidence has been accumulating that formation of this cluster in the kinesin‐microtubule complex allosterically stabilizes the CLOSED (hydrolysis‐competent) conformation of the active site switches and this point now seems clear. Cao et al.^8^ demonstrate that this is also the case for tubulin binding and that mutating the IKN triplet indeed inhibits both the tubulin‐activated and microtubule‐activated ATPases. A key point however is that the main effect of triple alanine mutagenesis of this IKN sequence is not to inhibit the microtubule‐activated ATPase, but rather to degrade the coupling between stepping and ATP‐turnover,^22^ so that futile cycles of ATP turnover occur. This suggests that in addition to a role in stabilizing the hydrolytically competent conformation of the active site, neck linker docking serves to minimize futile cycling, by stabilizing the TRAPPED K.ADP state of the motor domain, ensuring prompt detachment from the microtubule and tight coupling of stepping to ATP turnover.

## MICROTUBULE‐ACTIVATION OF ADP RELEASE

Microtubule binding powerfully activates the kinesin ATPase. The major effect of microtubule binding is on the rate constant for ADP release, which is rate limiting in the absence of microtubules and accelerated by ∼10^3^‐fold in their presence. In the kinesin mechanism there is an antagonism between MgADP trapping and microtubule binding, with each destabilizing the other. Effectively, kinesin motor domains in the absence of microtubules drop into an autoinhibited, TRAPPED K.ADP state, whilst microtubule binding triggers escape from this state by catalyzing ADP release. For some kinesins, unpolymerized tubulin also activates ADP release.^23^ How does microtubule binding accelerate ADP release? In an early review, Vale suggested that microtubules activate ADP release by accelerating Mg^2+^ release,^24^ by analogy with the action of GEFs on small G‐proteins. Nitta and colleagues^14^ showed that for Kif1A (kinesin‐3), high Mg^2+^ concentrations indeed inhibit ADP release from kinesin in both the presence and absence of microtubules. They suggested that a salt bridge forms between beta tubulin and the loop 7 of Kif1A, directly pulling open the Sw1 and releasing Mg^2+^. This mechanism may not be general, since the target residue on tubulin is not well‐conserved, but our current picture does lack a mechanism for microtubules to drive Mg^2+^ release and a more direct mechanism to pull the Sw1 away from the Mg^2+^ is attractive. An alternative, or perhaps complimentary process, would involve a twisting of the central beta sheet of kinesin. This is not evident in the kinesin‐tubulin apo structure, but an analogous process is important in myosin for withdrawing the Sw1 and releasing Mg^2+^. Modelling suggests that straightening the conformation of tubulin could promote beta sheet twisting in kinesin.^25^ Shang and colleagues^26^ using fitting into cryoEM reconstructions of the complex of kinesin with straight tubulin, highlight the role of a key “lynchpin” residue in H4, N255, in engaging and withdrawing the Sw1 and the release of Mg^2+^. Mutating this residue abrogates microtubule activation of the kinesin ATPase.^27^ The associated extension of the H4 helix is clearly stabilized by microtubule binding, but its first cause appears to be ATP binding, since it is also seen in the kinesin‐5.AMPPNP structure, in the absence of microtubules.^4^ An extended H4 is also sometimes seen in K.ADP crystal structures in the absence of MTs. In the K.ADP structure of *N. Crassa* kinesin‐1 (Nkin; 1G0J), the extended portion of H4 is stabilized by a salt bridge linking it to Sw1.

Prior to microtubule‐activation, trapping of MgADP by the motor domain stabilizes it in an auto‐inhibited weak binding state. It is possible that monastrol‐like drugs that bind to kinesin‐5 and stabilize its weak K.ADP state^28^ work by enhancing Mg^2+^ binding.

## PHOSPHATE RELEASE

The closure of Sw1 and Sw2 and their linkage by the critical salt bridge forms the phosphate tube, a blind‐ended cavity. As Figure 2 shows, the transition from CLOSED to TRAPPED remodels the Sw1, exposing the phosphate site and allowing phosphate to escape. The CLOSED to TRAPPED transition, which converts the motor from strong to weak binding, is coupled to phosphate release, allowing phosphate release to act as a gate^29^ controlling access to the TRAPPED state, and therefore controlling unbinding of the motor domain from the microtubule. The affinity of kinesin for free phosphate is in the tens of millimolar, so that phosphate rebinding^29^ can nonetheless occur at high phosphate concentration, and indeed rebinding of both phosphate and ADP can drive a (very) low rate of ATP resynthesis.^30^

## NECK LINKER DOCKING, FORCE GENERATION AND FORCE‐SENSING

**Figure 3:**
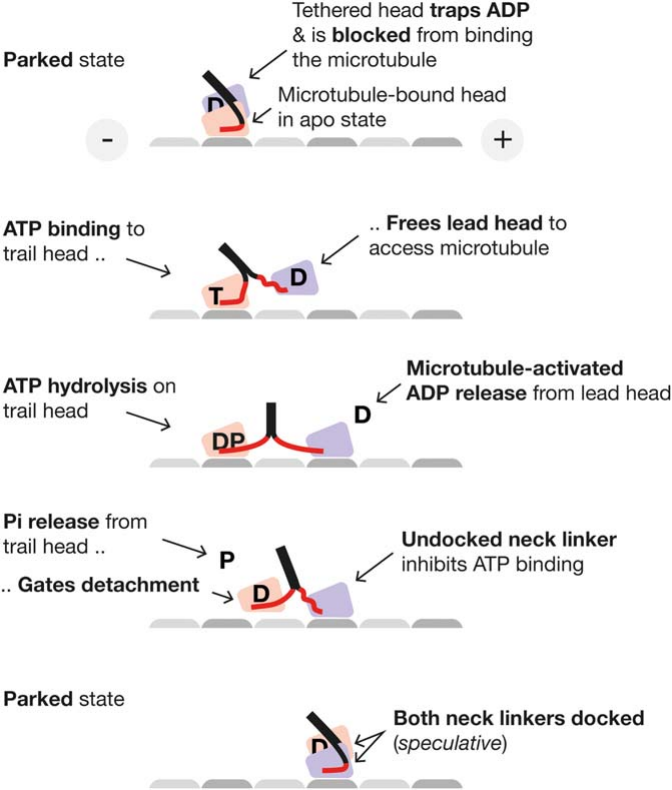
Neck linker docking and the coordination of molecular walking. The neck linkers (red) undergo a dock–undock cycle that feeds back on the active site conformations of the two motor domains (heads). Between steps, kinesin waits for ATP with one head (the trailing head) strongly bound to the microtubule track and the other (the leading, tethered head) held clear of the microtubule. Recent high resolution tracking work using gold nanobead labelling confirms that the tethered head is alongside the bound head in the wait‐ATP state.^40^, ^41^ The molecular mechanism by which the tethered head is prevented from binding both microtubules and free tubulin^23^ before ATP has bound to its partner remains unclear. In the scheme shown, docking of both neck linkers causes the tethered head to park against its partner. This is speculative.^23^ Whatever the mechanism constraining the tethered head, it is clear that the wait‐ATP state is pivotally important. As [ATP] increases, the time spent in the wait‐ATP state of each cycle reduces, but this does not diminish its importance ‐ even at low load and high ATP, the wait‐ATP gate serves to reset the phase‐lag between the two heads, by requiring that ATP bind to one head before the other can step. Once ATP binds to the trailing head, the tethered head rapidly locates and binds to its next site, with its diffusional search focused by neck linker docking on the trailing head. Microtubule binding triggers ADP release from the leading head, which is favored because its neck linker is undocked. Phosphate release from the trailing head gates its detachment in the K.ADP state, which is accelerated by docking of the neck linker on the trailing head. The coordination mechanism works well at zero load and under backwards load, but breaks down under forwards load.^29^, ^34^

Docking of the neck linker, a short, C‐terminal peptide (TIKNTVSVNELT) against the main part of the kinesin motor domain was originally proposed to act as a lever to generate force in response to ATP binding.^16^ It is very clear that the neck linker dock/undock cycle of kinesin‐1 is intimately coupled to, and required for, the generation of force and movement, but it remains unclear how much force and displacement is generated by neck linker docking itself. Measurements of the equilibrium constant for neck linker docking using spin probe labelling and EPR indicated that relatively little work (about 4 pN nm^31^) could be done, but the probe attached to the neck linker was necessarily bulky relative to the neck linker and could have influenced docking. The question of whether neck linker docking in the ATP state can generate sufficient force to account for kinesin's ability to step 8.2 nm against loads of up to 7.2 pN remains open. By contrast the evidence for a role for the neck linker in controlling and coordinating kinesin stepping (force‐sensing rather than force‐generation) is clear, as is the requirement for neck linker docking for force generation by surfaces of monomeric kinesin motor domains.^22^ Pulling backwards on a microtubule‐bound kinesin head will tend to undock its neck linker, whilst pulling forwards will favor docking. In walking kinesin dimers (Figure 3), this ability of the neck linker to sense the direction of the force is used to coordinate the ATPase cycles of the two coupled motor domains (“heads”). Backwards force on the leading head of a walking dimer will undock its neck linker and thereby inhibit ATP binding and hydrolysis, whilst forwards force on the trailing head will tend to dock its neck linker and stabilize ATP binding.

It seems probable that the same neck linker‐based force‐sensing mechanism can govern product ADP trapping as well as ATP hydrolysis. The structural and kinetic evidence suggests that neck linker docking stabilizes both the hydrolysis competent CLOSED K.ATP state of the active site and the subsequent TRAPPED K.ADP state. Neck linker docking is a key feature of the super‐inhibited K.ADP dimer state crystallized by Kaan and colleagues.^32^ By promoting neck linker undocking, backwards load would promote microtubule‐activated ADP release from the lead head of a walking dimer, and tend to inhibit ADP rebinding. This may be the basis of the direction‐dependent detachment rate constant of the K.ADP state.^33^ Meanwhile on the trail head, forwards load will favor neck linker docking with ADP in the active site, favoring prompt detachment in the TRAPPED state. Recent single molecule optical trapping work is revealing. Milic and colleagues^29^ (Pi) show beautifully that pulling forwards on a walking kinesin dramatically reduces its processive run length (the number of steps taken per processive run), consistent with premature trail head detachment, whilst adding back phosphate mitigates. Andreasson^34^ show using neck linker truncations that mechanical strain is not the key to coordinated stepping; rather, it is neck linker docking and undocking that matters. Dogan et al.^35^ apply force‐ramps to single tethered kinesin heads and demonstrate a strong dependence of the detachment force on the direction of the load, with backwards load (and neck linker undocking) stabilizing microtubule binding, and forwards load (and neck linker docking) destabilizing.

## CONCLUDING COMMENTS

Recently determined crystal structures of tubulin‐kinesin‐1 complexes represent a major achievement and milestone for the field and the foregoing discussion relies heavily on these structures. Nonetheless the tubulin in these structures is in a curved conformation that does not polymerize,^25^ and whilst some kinesins are activated by free tubulin,^23^ many are not. CryoEM structures of kinesin‐microtubule complexes are rapidly improving in resolution^26^, ^36^, ^37^ and we can confidently expect the field to converge on an atomic model of the entire kinesin structural cycle. Looking to the future, our ultimate goal must be to understand the structural cycle of kinesin whilst it is stepping under load. To achieve this, the field will need to continue to break new technical ground.
